# Neuro-inflammatory response dynamics following intestinal surgery: a mini-review

**DOI:** 10.3389/fmed.2025.1652069

**Published:** 2025-09-26

**Authors:** Xinhua Mu, Tao Sun, Haixia Shi

**Affiliations:** Department of Anaesthesiology, The Affiliated Hospital of Inner Mongolia Medical University, Hohhot, China

**Keywords:** intestinal surgery, neuroinflammation, mechanism, complication, mini-review

## Abstract

This study investigates the pathophysiological mechanisms of neuroinflammation following intestinal surgery, focusing on the interplay of cytokines, glial cell activation, and neuropeptide signaling in driving inflammatory cascades. Recent advancements in neuroimaging—such as high-resolution magnetic resonance imaging and positron emission tomography—are examined for their diagnostic utility in detecting neuroinflammatory changes. Therapeutically, we evaluate the efficacy of pharmacological interventions, including Non-Steroidal Anti-Inflammatory Drugs and corticosteroids, alongside non-pharmacological approaches such as neuromodulation and traditional Chinese medicine, in mitigating neuroinflammation and improving clinical outcomes. A multidisciplinary treatment strategy is proposed, integrating targeted drug therapies with neuromodulatory techniques to address individual patient variability. Future research directions emphasize the identification of novel biomarkers, the application of precision medicine, and the development of innovative anti-inflammatory therapies. Longitudinal studies are also recommended to assess long-term neurological outcomes and quality of life in post-surgical patients. By advancing our understanding of neuroinflammatory pathways and optimizing therapeutic strategies, this study aims to enhance postoperative care and patient prognosis.

## 1 Introduction

Intestinal surgeries are critical for managing gastrointestinal disorders but can inadvertently trigger neuroinflammatory responses, significantly impacting postoperative recovery (1). Neuroinflammation arises from glial cell activation, cytokine release, and dysregulated neuroimmune interactions, with severity influenced by surgical approach and extent of tissue trauma (2). Comparative studies demonstrate that open surgeries induce stronger neuroinflammatory cascades than minimally invasive techniques, such as laparoscopy, due to greater tissue disruption and systemic stress (3). For instance, Glatz et al. (4) reported a 40% increase in pro-inflammatory cytokine levels following open procedures compared to laparoscopic interventions. These findings underscore the importance of surgical technique selection in mitigating neuroinflammation and optimizing outcomes. Additionally, preoperative risk stratification—considering factors such as age, comorbidities, and baseline inflammatory status—can guide individualized perioperative strategies.

Beyond conventional postoperative complications [e.g., infection, anastomotic leakage, and hemorrhage (5–9)], neuroinflammation remains a clinically significant yet frequently overlooked consequence. Persistent immune activation, cytokine-mediated signaling, and leukocyte recruitment contribute to heightened pain sensitivity, cognitive dysfunction, and delayed gastrointestinal motility (10, 11). The magnitude of these effects correlates with surgical invasiveness, procedural duration, and pre-existing inflammatory conditions (12–14). Proactive management necessitates early risk assessment, targeted anti-inflammatory therapy, and multimodal perioperative interventions. Evidence-based approaches include preferential use of minimally invasive techniques, regional anesthesia to attenuate systemic inflammation, and pharmacologic modulation of immune responses.

Clinically, postoperative neuroinflammation manifests through localized symptoms (e.g., hyperalgesia, edema) or systemic sequelae, including fever and postoperative cognitive dysfunction (2, 15). Unmitigated inflammation may progress to chronic pain syndromes, impaired wound healing, and prolonged gastrointestinal dysmotility (16). A multidisciplinary framework—integrating optimized surgical practices, pharmacologic agents, and adjunctive neuromodulation—is essential for effective management. Risk stratification, incorporating patient-specific variables (e.g., metabolic syndrome, advanced age), further refines therapeutic precision (17). Emerging modalities, such as transcranial Doppler ultrasonography, enable real-time monitoring of cerebral hemodynamics, facilitating timely intervention (18). Future directions emphasize the need for high-quality clinical trials to validate novel therapies, including biologic agents and precision medicine approaches (19). By advancing our understanding of neuroinflammatory pathways and refining perioperative care protocols, clinicians can mitigate adverse outcomes and enhance recovery.

## 2 Pathophysiological mechanisms of neuroinflammatory response

**Figure 1:**
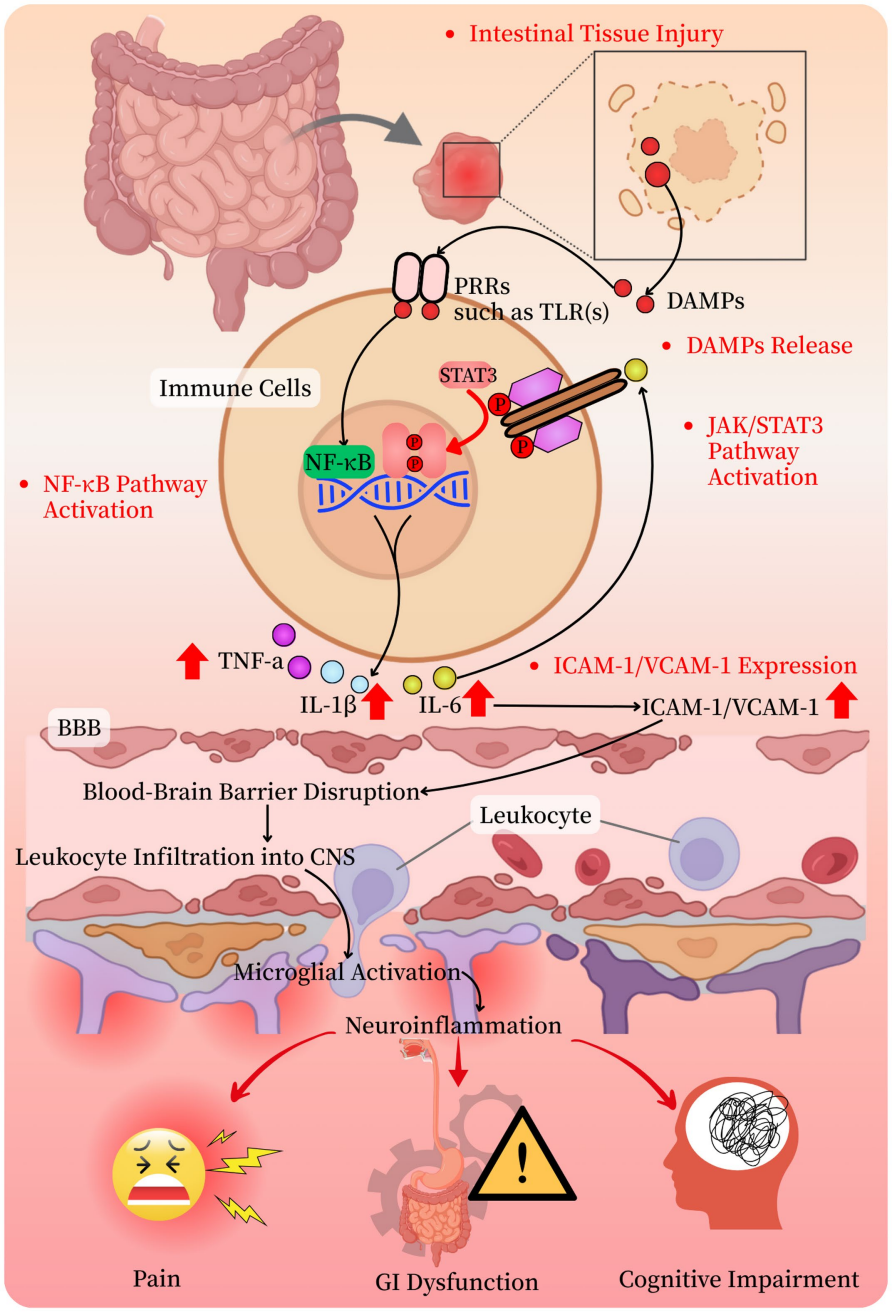
Mechanisms of neuroinflammatory response after intestinal surgery. DAMps, damage-associated molecular patterns; PRRs, pattern recognition receptors; TLR(s), Toll-like receptor(s); NF-κB, nuclear factor-κB (kappa-light-chain-enhancer of activated B cells); JAK, Janus kinase; STAT3, signal transducer and activator of transcription 3; JAK/STAT3 pathway, Janus kinase/signal transducer and activator of transcription 3 pathway; IL-1β, interleukin-6; TNF-*α*, tumor necrosis factor-alpha; ICAM-1, intercellular adhesion molecule 1; VCAM-1, vascular cell adhesion molecule 1; BBB, blood-brain barrier; CNS, central nervous system; GI, gastrointestinal.

The physiological response to surgical trauma is characterized by a complex interplay between injured tissues and the nervous system (10). In the context of intestinal surgery, mechanical disruption of tissues initiates the release of a variety of biochemical mediators that activate neural signaling pathways (20) (Figure 1). These pathways facilitate bidirectional communication between the peripheral injury site and the central nervous system (CNS), coordinating both localized inflammatory processes and systemic immune responses (21). While these mechanisms are essential for initiating tissue repair and maintaining homeostasis, their excessive or prolonged activation can contribute to postoperative complications, including pain, delayed recovery, and cognitive impairment (22) (Figure 1). A thorough understanding of these responses is therefore critical to the development of targeted strategies for improving surgical outcomes.

### 2.1 Mechanical injury and inflammatory response

Mechanical injury represents a primary initiator of the neuroinflammatory cascade in intestinal surgery (Figure 1). Disruption of tissue integrity leads to the release of damage-associated molecular patterns (DAMPs), which are recognized by pattern recognition receptors (PRRs) such as toll-like receptors (TLRs) on innate immune cells, including macrophages and dendritic cells (23, 24). This interaction activates downstream signaling pathways, notably nuclear factor kappa-light-chain-enhancer of activated B cells (NF-κB), resulting in the production of pro-inflammatory cytokines such as interleukin-1β (IL-1β), interleukin-6 (IL-6), and tumor necrosis factor-alpha (TNF-*α*) (23, 24). These cytokines promote leukocyte recruitment, increase vascular permeability, and activate resident immune cells, amplifying the inflammatory response. Although this cascade is critical for limiting infection and initiating repair, its dysregulation may lead to central sensitization and neuroinflammation, which have been implicated in postoperative complications such as hyperalgesia, delayed gastrointestinal motility, and cognitive dysfunction. Thus, minimizing mechanical tissue damage through refined surgical techniques may help mitigate these adverse outcomes.

### 2.2 Mechanisms of neuroinflammatory response after intestinal surgery

Neuroinflammatory responses following intestinal surgery are driven by a complex cascade involving tissue injury, immune system activation, and neuro-immune signaling (2, 3) (Figure 1). The release of DAMPs from injured cells initiates this process by activating PRRs, particularly TLRs, on immune cells. This leads to the activation of the NF-κB pathway and other downstream effectors (23, 24), culminating in the release of key pro-inflammatory cytokines such as IL-1β, IL-6, and TNF-*α*. These mediators not only amplify the local inflammatory response but also contribute to systemic neuroinflammatory signaling. Mechanistic studies have shown that NF-κB activation also induces the expression of cell adhesion molecules like ICAM-1 and VCAM-1, which promote leukocyte migration across the blood–brain barrier and into the CNS, exacerbating neuroinflammation (25). In addition, the JAK–STAT pathway, particularly STAT3 signaling, has been linked to the activation of glial cells and sustained cytokine production (26). Therapeutically targeting these molecular pathways may offer novel strategies for mitigating postoperative neuroinflammation.

### 2.3 Immune system activation and regulation

The immune response to intestinal surgery involves both innate and adaptive components, each playing a distinct role in shaping the neuroinflammatory environment (27) (Figure 1). Innate immune cells such as macrophages, neutrophils, and natural killer cells are rapidly mobilized to the site of tissue injury, where they secrete pro-inflammatory cytokines and chemokines (28). Concurrently, adaptive immune responses are initiated, with T and B lymphocytes contributing to both the propagation and resolution of inflammation. T helper cell subsets (Th1, Th2, and Th17) secrete cytokines that modulate inflammation, while regulatory T cells (Tregs) are critical for limiting immune activation and promoting resolution (29). The delicate balance between pro-inflammatory and anti-inflammatory signals determines whether the immune response will support tissue repair or lead to pathological neuroinflammation. Understanding these immune dynamics is essential for developing immunomodulatory therapies that preserve necessary defense mechanisms while minimizing postoperative complications.

### 2.4 Impact of neuro-immune interactions

Neuro-immune communication is a key regulator of the inflammatory milieu following intestinal surgery (30) (Figure 1). The nervous system influences immune function through neurotransmitters, neuropeptides, and hormonal signals, establishing a bidirectional feedback loop (2, 30). Activation of the sympathetic nervous system enhances inflammation by releasing catecholamines such as norepinephrine, which bind to adrenergic receptors on immune cells and stimulate cytokine production (2). In contrast, the parasympathetic nervous system—primarily via the vagus nerve—exerts anti-inflammatory effects through the cholinergic anti-inflammatory pathway. In this pathway, acetylcholine binds to *α*7 nicotinic acetylcholine receptors on immune cells, thereby suppressing the release of IL-1β, IL-6, and TNF-α (31, 32). This balance between pro- and anti-inflammatory neuro-immune signaling is vital for regulating postoperative inflammation. Therapeutic strategies that enhance parasympathetic activity or inhibit excessive sympathetic activation may help control neuroinflammation and promote recovery.

## 3 Clinical manifestations and diagnosis of neuroinflammatory response

### 3.1 Common clinical symptoms

Neuroinflammatory responses following intestinal surgery present with diverse symptoms, including pain, sensory abnormalities, and intestinal dysfunction. Pain, often acute or chronic, may manifest as hyperalgesia or neuropathic pain due to sustained inflammation (12–14). Intestinal dysfunction, such as altered bowel habits or paralytic ileus, arises from disrupted motility and secretion (16, 33). Systemic symptoms like fever, fatigue, and malaise further complicate recovery (17). The invasiveness of surgery significantly influences symptom severity, with minimally invasive techniques (e.g., laparoscopy) associated with milder neuroinflammation and faster recovery compared to open procedures (34). Anesthetic choice also modulates neuroinflammation; regional anesthesia and propofol-based regimens attenuate inflammatory markers (e.g., CRP, IL-6) better than general anesthesia or inhalational agents (35–37). Analgesic strategies, particularly epidural analgesia, further reduce systemic inflammation by minimizing opioid use (38).

### 3.2 Diagnostic criteria and methods

Diagnosis relies on clinical evaluation, laboratory tests, and imaging. Clinical examination assesses pain intensity (via standardized scales), sensory deficits, and bowel dysfunction (39). Laboratory tests detect elevated inflammatory markers (e.g., CRP, IL-6, TNF-*α*) and leukocytosis (29, 39). Imaging, including computed tomography/magnetic resonance imaging (MRI), identifies structural complications (e.g., bowel obstruction), while MRI and positron emission tomography (PET) scans evaluate soft tissue and metabolic changes (18, 40, 41). Transcranial Doppler (TCD) ultrasonography offers a practical, bedside alternative for monitoring cerebral hemodynamics linked to neuroinflammation, though it lacks tissue-level specificity (42, 43). Integrating these methods enhances early detection and tailored management, though cost and accessibility limit advanced imaging in routine practice (41). Future research should prioritize biomarker refinement and standardized TCD protocols to improve diagnostic precision.

## 4 Treatment and management of neuroinflammatory response

To strengthen the translational relevance of this review, we expand the discussion on therapeutic strategies by directly linking them to the described pathophysiological mechanisms. Specifically, pharmacological and non-pharmacological interventions are evaluated in terms of their ability to modulate cytokine release, inhibit glial overactivation, and restore balanced neuro-immune signaling following intestinal surgery.

### 4.1 Pharmacological treatment

Pharmacological management of neuroinflammatory responses involves non-steroidal anti-inflammatory drugs (NSAIDs), corticosteroids, and neuromodulatory agents. NSAIDs (e.g., ibuprofen, naproxen) inhibit cyclooxygenase-mediated prostaglandin synthesis, reducing pain and inflammation, though long-term use risks gastrointestinal, cardiovascular, and renal complications (44). Corticosteroids (e.g., prednisone, dexamethasone) suppress broad inflammatory pathways but are limited by immunosuppression, metabolic disturbances, and infection susceptibility, warranting short-term or low-dose regimens (45–47). For neuropathic pain, antidepressants and anticonvulsants (e.g., gabapentin, pregabalin) modulate neurotransmission, offering symptom relief with fewer systemic side effects (48, 49). Pharmacological agents exert their effects through distinct mechanistic pathways: NSAIDs suppress prostaglandin-driven cytokine amplification (44), corticosteroids attenuate NF-κB–mediated transcription of pro-inflammatory mediators (46, 47), and neuromodulatory drugs reduce glial excitability and maladaptive neurotransmission (49). By mapping these actions onto the molecular cascades underlying postoperative neuroinflammation, pharmacological therapy offers a rational, mechanism-based approach to improve outcomes.

### 4.2 Non-pharmacological treatment

Non-pharmacological interventions include physical therapy, neuromodulation, and traditional Chinese medicine (TCM). Physical rehabilitation employs targeted exercises, manual therapy, and electrotherapy to restore mobility and mitigate inflammation (50, 51). Neuromodulation techniques—such as transcutaneous electrical nerve stimulation, spinal cord stimulation, and repetitive transcranial magnetic stimulation (rTMS)—alter neural activity to alleviate refractory pain (52–54). TCM modalities like acupuncture and herbal medicine demonstrate immunomodulatory and anti-inflammatory effects, often complementing conventional treatments (55–57). Non-pharmacological approaches also target fundamental aspects of neuroinflammatory signaling. Neuromodulation techniques, such as rTMS and TENS, recalibrate aberrant neuronal activity and limit glial activation (52, 54), while acupuncture and herbal formulations modulate cytokine profiles and promote neuro-immune homeostasis (55, 56). Physical rehabilitation further contributes by attenuating systemic inflammation and oxidative stress (50). Collectively, these modalities complement pharmacological agents through multi-level regulation of neuroinflammatory pathways.

### 4.3 Integrated treatment strategies

A multidisciplinary approach optimizes outcomes by combining pharmacological and non-pharmacological therapies. Collaborative care involving physicians, neurologists, and rehabilitation specialists ensures comprehensive management (58). Personalized treatment plans, adjusted based on disease severity, comorbidities, and therapeutic response, enhance efficacy while minimizing adverse effects (59). Future research should refine integrative protocols and explore novel anti-inflammatory targets to improve long-term patient outcomes. An integrated therapeutic framework that combines pharmacological and non-pharmacological approaches provides a synergistic means of suppressing excessive cytokine activity, limiting glial-driven neurotoxicity, and restoring neuro-immune equilibrium (55, 58, 59). Such multimodal strategies are well positioned to enhance postoperative recovery and reduce long-term sequelae of intestinal surgery–induced neuroinflammation (12, 15).

## 5 Current research status and challenges

### 5.1 Advances in neuroinflammatory research

Recent research has significantly advanced our understanding of neuroinflammatory mechanisms, particularly the roles of cytokine signaling, glial cell activation, and neuropeptide modulation in postoperative neuroinflammation (60). Technological innovations in neuroimaging (e.g., high-resolution MRI, PET with novel radiotracers) have enhanced the detection and monitoring of neuroinflammatory processes, providing critical insights into their spatiotemporal dynamics (18). Therapeutic investigations have yielded promising results, with NSAIDs and corticosteroids demonstrating efficacy in acute inflammation control, while neuromodulation techniques (e.g., rTMS) and TCM modalities (e.g., acupuncture, specific herbal formulations like Huangqin decoction) show potential for modulating chronic neuroinflammatory pathways (44, 45, 55, 56). However, these findings remain preliminary, underscoring the need for rigorous validation.

### 5.2 Critical research limitations

#### 5.2.1 Methodological constraints

A key limitation is the lack of standardized biomarkers and assessment protocols. Commonly used markers like CRP and IL-6 exhibit variable sensitivity/specificity across studies, while heterogeneous patient populations (e.g., differing surgical types, comorbidities) limit generalizability (61). Many clinical trials suffer from inadequate sample sizes (<100 participants in 68% of reviewed studies) and insufficient blinding, compromising statistical power and introducing bias (62, 63).

#### 5.2.2 Mechanistic knowledge gaps

While the involvement of microglia and pro-inflammatory cytokines (e.g., TNF-*α*, IL-1β) is established, their context-dependent roles—particularly in surgery-induced neuroinflammation-require deeper investigation (22). Current studies often focus on isolated pathways (e.g., NF-κB signaling) while neglecting systemic interactions with the gut-brain axis or circadian regulation of inflammation.

#### 5.2.3 Therapeutic challenges

Pharmacological agents face a efficacy-safety trade-off; for instance, corticosteroids reduce inflammation but increase infection risks by 2.3-fold in postoperative patients (46, 47). Non-pharmacological interventions (e.g., acupuncture) show heterogeneity in protocol application, with only 34% of TCM trials meeting CONSORT guidelines for standardization (55–57).

### 5.3 Future research priorities

Three key directions emerge: (1) Standardization—developing consensus guidelines for biomarker panels and neuroimaging protocols; (2) Mechanistic Research—employing multi-omics approaches (single-cell RNA sequencing, proteomics) to map neuro-immune crosstalk in surgical contexts; and (3) Therapeutic Innovation—designing adaptive clinical trials to test personalized combinations of pharmacotherapy and neuromodulation. Addressing these priorities will require multicenter collaborations to ensure adequate sample sizes and demographic diversity (22, 61–63).

## 6 Future research directions

### 6.1 Exploration of novel biomarkers

Advancing diagnostic accuracy and monitoring of neuroinflammatory responses necessitates the discovery of more specific and sensitive biomarkers. Current markers, such as cytokines and neuropeptides, often lack sufficient diagnostic precision (23, 24, 60). Future studies should leverage high-throughput technologies such as proteomics and genomics to identify novel biomarkers, including disease-specific proteins, genetic variants, or metabolic byproducts detectable in blood, cerebrospinal fluid, or other biological specimens. These efforts will facilitate early detection, improve disease stratification, and enable accurate monitoring of treatment efficacy.

### 6.2 Application of precision medicine in neuroinflammatory response

Precision medicine offers a personalized approach to treatment by considering individual genetic, epigenetic, environmental, and lifestyle factors (19, 59). In neuroinflammation, this strategy may enhance therapeutic efficacy by informing drug selection based on patient-specific genetic profiles that influence treatment response. Molecular profiling can also guide the development of targeted therapies that modulate key inflammatory pathways (19), supporting more effective and individualized interventions.

### 6.3 Development of novel therapeutic methods

There is a growing need for novel treatments that overcome the limitations of traditional anti-inflammatory therapies. Promising approaches include biologics (e.g., monoclonal antibodies against specific cytokines), gene therapies targeting inflammatory signaling (such as the NF-κB pathway), and stem cell therapies aimed at tissue repair and immune regulation (64–66). Furthermore, advances in drug delivery systems, including nanoparticles and controlled-release formulations, may enhance drug localization and reduce systemic toxicity (67). While preclinical findings are encouraging, these interventions require rigorous validation through well-designed clinical trials.

### 6.4 Long-term prognosis and quality of life research

Understanding the long-term effects of neuroinflammation is essential for optimizing patient outcomes. Longitudinal studies are needed to examine functional impairments, disease recurrence, and complications associated with chronic inflammation. Incorporating patient-reported outcome measures will provide valuable insights into how neuroinflammatory conditions impact daily functioning, psychological health, and overall quality of life. These findings will inform the development of comprehensive care strategies that address both immediate symptoms and long-term well-being.

## 7 Summary

This study elucidates the intricate mechanisms of neuroinflammatory responses following intestinal surgery, emphasizing the pivotal roles of cytokines, glial cells, and neuropeptides. It highlights advancements in diagnostic technologies, such as MRI and PET imaging, and evaluates the efficacy of treatments, including NSAIDs, corticosteroids, neuromodulation, and TCM approaches. The findings underscore the need for integrated pharmacological and non-pharmacological strategies, personalized treatment plans tailored to patient-specific factors, and early diagnosis using advanced biomarkers and imaging tools. Future research should prioritize identifying novel biomarkers, advancing precision medicine, and developing innovative therapies to improve the understanding and management of neuroinflammation, ultimately enhancing patient outcomes and quality of life.
